# Self-Reported Social Determinants of Health and Area-Level Social Vulnerability

**DOI:** 10.1001/jamanetworkopen.2024.12109

**Published:** 2024-05-20

**Authors:** Emily Brignone, Keith LeJeune, Amanda E. Mihalko, Amy L. Shannon, Lawrence I. Sinoway

**Affiliations:** 1 Highmark Health Research Institute, Pittsburgh, Pennsylvania; 2 Allegheny Health Network Research Institute, Pittsburgh, Pennsylvania; 3 Social Determinants of Health Department, Highmark Health, Pittsburgh, Pennsylvania; 4 Heart and Vascular Institute, Penn State College of Medicine, Hershey, Pennsylvania

## Abstract

**Importance:**

Many health care systems are investing resources in identifying social determinants of health (SDoH) needs and facilitating interventions among the populations they serve. Because self-reported SDoH information is lacking, area-level measures are often used to estimate needs and direct resources.

**Objective:**

To describe the large-scale deployment of SDoH assessments by a health system and determine the extent to which self-reported SDoH needs identified therein are associated with census tract–level social vulnerability measured using the Social Vulnerability Index (SVI).

**Design, Setting, and Participants:**

This cross-sectional study assessed SDoH needs between January 1, 2020, and April 30, 2023, in both payer and clinical care settings. Modalities included telephonic outreach, face-to-face clinical interactions, self-entry into a tablet or kiosk, and web-based survey tools. Participants included individuals who responded to the assessment and had sufficient information for census tract identification. Respondents included both Highmark Health Plan members and nonmembers. Health plan members responded to the assessment through health plan programs or platforms, and both members and nonmembers responded to assessments during inpatient or outpatient encounters with the affiliated health system.

**Main Outcomes and Measures:**

Overall and domain-specific SDoH needs self-reported through assessments, and severity and complexity of needs identified. Residential social vulnerability measures included overall SVI and the 4 conceptual themes comprising overall SVI.

**Results:**

In total, 841 874 assessments were recorded for 401 697 individuals (55.1% women; median [IQR] age, 55 [41-70] years). Social determinants of health needs were identified in 120 769 assessments (14.3%). Across all SDoH domains, increasing SVI was associated with a higher positivity rate (eg, 11.2% of those residing in the lowest-risk SVI quintile reported a need compared with 22.7% among those residing in the highest-risk quintile). Associations varied by SDoH domain and SVI theme. After adjusting for demographic and screening characteristics, odds of positive screening among those residing in the highest-risk SVI quintile were 1.74 (95% CI, 1.62-1.86) to 3.73 (95% CI, 3.48-4.00) times the odds among those residing in lowest risk quintile.

**Conclusions and Relevance:**

In this cross-sectional study, the overall level of SDoH needs generally corresponded to area-level vulnerability. Some SDoH domains appeared far more sensitive to community characteristics than others. Notably, even among individuals from the highest-risk areas, the positive screening rate was roughly 1 in 4. These findings underscore the importance of individual-level SDoH data for service provision planning and health services research.

## Key Points

**Question:**

Are self-reported social determinants of health (SDoH) needs associated with area-level social vulnerability as measured by the Social Vulnerability Index (SVI)?

**Findings:**

This cross-sectional study of 841 874 assessments in 401 697 individuals found that self-reported SDoH needs were positively associated with SVI, with socioeconomic and racial and ethnic minority status themes most strongly associated with self-reported needs across all domains. Targeting individual SDoH needs based on SVI is likely to yield many false-positive results.

**Meaning:**

While self-reported SDoH needs are generally more prevalent in socially vulnerable areas, individual-level data can enhance service provision planning and research.

## Teaser

This cross-sectional study compares self-reported social determinants of health needs and Social Vulnerability Index associated with the individual’s residence among members and nonmembers of a large health care system in Pennsylvania.

## Introduction

According to the World Health Organization, social determinants of health (SDoH) “are the nonmedical factors that influence health outcomes. They are the conditions in which people are born, grow, work, live, and age, and the wider set of forces and systems shaping the conditions of daily life.”^1^ These factors include socioeconomic status, health behaviors, health care access and quality, and the physical environment. They contribute up to 80% to a patient’s quality of life and life expectancy^2^ and exist at multiple interacting levels (eg, individual, family, community) to influence health outcomes.^3^ The social ecological model of health provides a useful framework for understanding how these factors interact to create health risks or protective factors, which can identify individuals and communities at risk.

In 1989, the Centers for Disease Control and Prevention created the Social Vulnerability Index (SVI) to identify populations at risk of harm in response to natural disasters.^4^ The SVI uses 15 census measures to estimate vulnerability using 4 themes: socioeconomic status, household characteristics, racial and ethnic minority status, and housing type and transportation.^5^ The SVI has been used to explain and project infectious disease spread^6,7^ and demonstrate the importance of social vulnerability in risk for cardiometabolic conditions,^8^ cancer,^9^ and chronic lung disease mortality.^10^

Recognizing the importance of community-level SDoH factors in health outcomes is essential for understanding and solving social needs. However, in the health care setting, community-level measures can be challenging to integrate into clinical care and service provision. Inferring individual circumstances using community averages could lead to ecological fallacy and misallocation of resources. For example, well-resourced patients without SDoH needs may be targeted for outreach based on their address. Conversely, vulnerable patients residing in low-risk communities may be missed. Nonetheless, because individual-level data have not been available until recently, SVI is often used in its absence to estimate individual needs. While assessing individuals for SDoH needs is becoming more common, large-scale screening programs require significant investments in infrastructure, training, and time. Thus, coverage is still often poor, with self-reported data unavailable for most individuals.^11^

In 2019, Highmark Health, a national health and wellness organization, established a team to develop an SDoH assessment tool. The team included clinical case managers, project managers, information technology representatives, and executive and physician leadership from an affiliated health system, Allegheny Health Network. Clinically validated screening questions were used to create a 13-question assessment covering SDoH needs across several domains. The assessment was implemented in phases across multiple payer and clinical settings, with most assessments initiated by a staff member during face-to-face or telephonic interactions.

In this study, we compared self-reported SDoH needs and SVI associated with the individual’s residence. Drawing on the social ecological model, we hypothesized that composite SVI would be positively associated with self-reported needs, and SVI component themes would be most closely associated with SDoH needs in corresponding domains. We also hypothesized that associations would vary by theme and SDoH domain, with stronger associations among economic measures. We expect findings to underscore the importance of individualized assessments and guide their implementation, while informing the application and interpretation of community-level data in their absence.

## Methods

### Research Design and Population

The full retrospective dataset included 841 874 SDoH assessments administered in the payer and clinical care settings between January 1, 2020, and April 30, 2023. Observations represent unique assessments. Observations were excluded when the census tract could not be identified (n = 11 642) or when age or sex information was missing (n = 1446), with 11 776 (1.4%) excluded overall due to overlap. Respondents included Highmark Health Plan members who answered 1 or more payer-initiated assessment questions through a health plan program or platform, and both health plan members and nonmembers who answered 1 or more clinician-initiated assessment questions during an inpatient or outpatient encounter with the affiliated health system.

Most assessments were recorded in the clinical setting (676 815 [80.4%]). Individuals assessed multiple times during this period contributed multiple observations. Because the planned regression analyses assume independence of observations (ie, observations that are not related to one another), we created a secondary dataset of independent observations by randomly selecting exactly 1 assessment instance per unique respondent (n = 401 697). The study was approved by the institutional review board of the Allegheny Health Network Research Institute (acting for Highmark Health). Informed consent was waived because person-level records in the study were drawn from administrative data that were originally collected for other purposes, with no feasible method of retroactively obtaining consent and no risk of identifiability in published results. We followed the Strengthening the Reporting of Observational Studies in Epidemiology (STROBE) reporting guideline.

### Measures

Assessments of SDoH were captured using multiple modalities, including telephonic outreach, face-to-face clinical interactions, self-entry into tablets or kiosks during clinical encounters, and online survey tools. Clinic-based assessments were documented in electronic medical records, while payer-based assessments were recorded in corporate databases. A member-to-patient identifier crosswalk file was used to uniquely identify respondents across sources. All data sources were aggregated into a single working dataset.

The assessment included 13 questions covering 7 domains (social connections, health literacy, financial resource strain, transportation, food insecurity, safety, and housing stability). Table 1 lists questions with their sources and response options, including which responses indicate need. All questions included a decline option. Decline responses were counted as assessed with no need identified. Questions without responses were marked as missing. For the 806 862 assessments recorded in face-to-face or telephonic encounters (95.8% overall), a missing response indicated that the question was not asked. For the remaining digital self-entry assessments, missing responses indicate that the respondent skipped the question. Question-level completion rates and the share of declined are provided in Table 1. Calculations for domain-specific SDoH needs were based on the subset of assessments that included at least 1 response pertaining to the given domain. In addition to needs by domain, indicators for overall and combined SDoH needs were calculated, including a composite score ranging from 0 to 58 that used a scoring rubric to summarize aggregate severity and complexity of SDoH needs across all domains (eTable 1 in Supplement 1).

**Table 1.** Social Determinants of Health Assessment Questions and Sources and Descriptive Statistics for Item Completion, Declining to Respond, and Responses Indicating Need

Domain (question source)	Response options	No. (%) of assessments	
		Any response^a^	Chose not to answer^b^
Social connections (UCLA Loneliness Screener)^12^			
How often do you feel that you lack companionship?	Hardly ever; some of the time; often^c^; I choose not to answer	650 961 (77.3)	17 134 (2.6)
How often do you feel left out?	Hardly ever; some of the time; often^c^; I choose not to answer	646 684 (76.8)	16 489 (2.5)
How often do you feel isolated from others?	Hardly ever; some of the time; often^c^; I choose not to answer	646 438 (76.8)	16 468 (2.5)
Transportation needs (Protocol for Responding to and Assessing Patient Assets, Risks, and Experiences)^13^			
Has a lack of transportation kept you from medical appointments, meetings, work, or from getting things needed for daily living? Check all that apply.	No; yes, it has kept me from nonmedical meetings, appointments, work, or from getting things that I need^c^; yes, it has kept me from medical appointments or from getting my medications^c^; I choose not to answer	704 754 (83.7)	5199 (0.7)
Financial resource strain (Organization for Economic Co-operation and Development)^14^			
Sometimes people find that their income does not quite cover their living costs. In the last 12 mo, has this happened to you?	No; yes^c^; do not know; not applicable; I choose not to answer	674 578 (80.1)	11 506 (1.7)
Health literacy (Single-Item Literacy Screen)^15^			
How often do you need to have someone help you when you read instructions, pamphlets, or other material from your doctor or pharmacist?	Never; rarely; sometimes; often^c^; always^c^; I choose not to answer	683 833 (81.2)	8401 (1.2)
Food insecurity (Children’s Health Watch Hunger Vital Signs/American Academy of Family Physicians)^16^			
Within the past 12 mo we worried whether our food would run out before we got the money to buy more	Never true; sometimes true^c^; often true^c^; I choose not to answer	756 211 (89.8)	9976 (1.3)
Within the past 12 mo the food we bought didn’t last and we didn’t have money to get more	Never true; sometimes true^c^; often true^c^; I choose not to answer	748 667 (88.9)	10 176 (1.4)
Housing stability (Veteran’s Health Administration 2-item Universal Homelessness Screen)^17^			
In the past 2 mo, have you been living in stable housing that you own, rent, or stay in as part of a household?	No^c^; yes; I choose not to answer	731 878 (86.9)	8206 (1.1)
In the next 2 mo, are you worried that you may not have stable housing that you own, rent, or stay in as part of a household?	No; yes^c^; I choose not to answer	725 872 (86.2)	8906 (1.2)
Housing stability–utilities (Centers for Medicare & Medicaid Services Accountable Health Communities Screening Tool)^18^			
In the past 12 mo has the electric, gas, oil, or water company threatened to shut off services in your home?	No; yes^c^; already shut off^c^; I choose not to answer	667 538 (79.3)	8463 (1.3)
Safety			
Do you feel safe in your neighborhood?	No^c^; yes; I choose not to answer	657 886 (78.1)	11 593 (1.8)
Are you afraid of anyone close to you?	No; yes^c^; I choose not to answer	649 838 (77.2)	13 289 (2.0)

^a^ Based on all assessments recorded (N = 841 874).

^b^ Based on assessments with nonmissing responses for the given question.

^c^ Indicates a positive response.

Person-level characteristics were drawn from claims and clinical data, including age, sex, race (categorized as American Indian or Alaska Native, Asian, Black, Native Hawaiian or Other Pacific Islander, White, other, or unknown), ethnicity (categorized as Hispanic or Latino, non-Hispanic or non-Latino, or unknown), and address at time of assessment. Race and ethnicity data were collected because of their interrelated nature with social vulnerability, health care utilization, and health outcomes. Addresses were geocoded to identify census tracts, which served as the joining key for SVI data. In addition to raw scores and percentile ranks in the SVI, overall and theme vulnerability scores were divided into 5 quintiles using the national distribution of tract-level scores. Five quintiles were found to capture meaningful variability across the distribution of scores while providing ease of interpretation.

### Statistical Analysis

Descriptive statistics were computed for study variables and stratified by overall screening result (any vs no SDoH need) for the full dataset and for the independent observations subset. Because our large sample produced statistically significant results even at small effect sizes, *P* values were omitted from descriptive summaries.

Rates of positive assessments were computed for each SDoH domain and stratified by quintile for composite SVI and each component theme. Using the independent observations data subset (ie, 1 assessment per respondent), we computed a series of regression models estimating risk for a positive assessment in each domain as a function of SVI and several covariates. Covariates were selected based on hypothesized confounding and included age, sex, and assessment source. Race and ethnicity were summarized for context but not used in models, as roughly 30% of observations were missing not at random.

Univariate logistic regression was first used to estimate risk for a positive assessment overall and by domain as a function of SVI. Next, multivariate logistic regression models were used to additionally adjust for age, sex, and assessment source. Model estimates were computed in 2 ways to illustrate associations between independent variables and responses: first, as odds ratios (ORs) representing the estimated increase in positive assessment risk corresponding to a 1-U increase in SVI quintile; and second, as ORs representing the estimated increase in risk for a positive assessment, comparing the highest risk group (quintile 5) with the lowest risk group.

Because logistic regression models assumed that the association between SVI and the outcome was consistent across all levels, additional robustness tests were conducted using generalized additive models (GAM). These determined whether more complex models accounting for nonlinear associations between SVI and the log odds of a positive assessment produced meaningfully different results. The GAM inputs were identical to logistic regression model inputs, except SVI was measured in percentile ranks rather than quintiles to allow for the capture of full measure variability. Smoothing functions were included for SVI and age. To interpret and compare results from GAMs to logistic regression results, marginal contrasts transformed to the response scale were computed to produce ORs for intervals approximating the quintiles used in logistic regression models. Specifically, ORs were estimated across each 20% increment of the SVI distribution to mirror the estimated effect of a 1-U increase in quintile. Because this involves a large number of statistical comparisons, the Holm method of *P* value correction was implemented, with 2-sided *P* < .05 indicating statistical significance. All statistical analyses were completed in R, version 4.0.0 (R Project for Statistical Computing).

## Results

Overall, 841 874 assessments were recorded for 401 697 unique individuals (Table 2). The respondent population included 180 545 men (44.9%) and 221 152 women (55.1%) with a median (IQR) age of 55 (41-70) years. In terms of race, 342 respondents (0.1%) were American Indian or Alaska Native, 2915 (0.7%) were Asian, 20 034 (5.0%) were Black, 153 (0.03%) were Native Hawaiian or Other Pacific Islander, 281 090 (70.0%) were White, 3760 (0.9%) were of other race, and 93 403 (23.3%) were of unknown race. In terms of ethnicity, 3365 respondents (0.8%) were Hispanic or Latino, 295 299 (73.5%) were non-Hispanic or non-Latino, and 103 033 (25.6%) were of unknown ethnicity. The mean overall SVI for the cohort was in the 35th percentile, indicating that on average, the study population resided in communities somewhat less vulnerable than the national average. Individuals reporting SDoH needs were more likely to live in neighborhoods with above-average SVI than those reporting no needs (35.1% vs 24.9%). Needs were identified in 120 769 assessments (14.3%), with little variation in overall positivity rates during the collection period. The most commonly reported needs were financial resource strain (44 187 [6.6%]), housing instability (29 579 [4.0%]), health literacy (29 662 [4.3%]), and social connections (22 803 [3.5%]). Among positive assessments, 35 172 (29.1%) indicated needs in multiple domains.

**Table 2.** Descriptive Statistics for Social Determinants of Health Assessments and Person-Level Cohort

Characteristic	No. (%) of assessments			No. (%) of respondents^a^		
	Overall (N = 841 874)	No need identified (n = 721 105)	Need(s) identified (n = 120 769)	Overall (N = 401 697)	No need identified (n = 342 341)	Need(s) identified (n = 59 156)
Setting						
Clinical	676 815 (80.4)	597 363 (82.8)	79 452 (65.8)	273 773 (68.2)	245 749 (71.8)	28 024 (47.4)
Payer	165 059 (19.6)	123 742 (17.2)	41 317 (34.2)	127 924 (31.8)	96 792 (28.3)	31 132 (52.6)
Modality						
Face-to-face	648 041 (77.0)	575 904 (79.9)	72 137 (59.7)	259 479 (64.6)	234 828 (68.6)	24 651 (41.7)
Telephonic	158 821 (18.9)	120 326 (16.7)	38 495 (31.9)	122 445 (30.5)	93 790 (27.4)	28 655 (48.4)
Digital self-report	35 012 (4.2)	24 875 (3.4)	10 137 (8.4)	19 773 (4.9)	13 923 (4.1)	5850 (9.9)
Year						
2020	161 548 (19.2)	138 426 (19.2)	23 122 (19.1)	74 035 (18.4)	61 486 (18.0)	12 549 (21.2)
2021	243 517 (28.9)	209 340 (29.0)	34 177 (28.3)	109 137 (27.2)	93 157 (27.2)	15 980 (27.0)
2022	305 913 (36.3)	260 115 (36.1)	45 798 (37.9)	151 255 (37.7)	128 996 (37.7)	22 259 (37.6)
2023	130 896 (15.5)	113 224 (15.7)	17 672 (14.6)	67 270 (16.7)	58 902 (17.2)	8368 (14.1)
Race						
American Indian or Alaska Native	687 (0.1)	559 (0.1)	128 (0.1)	342 (0.1)	287 (0.1)	55 (0.1)
Asian	4544 (0.5)	3970 (0.6)	574 (0.5)	2915 (0.7)	2538 (0.7)	377 (0.6)
Black	43 091 (5.1)	35 844 (5.0)	7247 (6.0)	20 034 (5.0)	16 529 (4.8)	3505 (5.9)
Native Hawaiian or Other Pacific Islander	253 (0.03)	217 (0.03)	36 (0.03)	153 (0.03)	131 (0.03)	22 (0.04)
White	534 682 (63.5)	475 269 (65.9)	59 413 (49.2)	281 090 (70.0)	248 881 (72.7)	32 209 (54.4)
Other^b^	6212 (0.7)	5304 (0.7)	908 (0.8)	3760 (0.9)	3203 (0.9)	557 (0.9)
Unknown	252 405 (30.0)	199 942 (27.7)	52 463 (43.4)	93 403 (23.3)	70 972 (20.7)	22 431 (37.9)
Ethnicity						
Hispanic or Latino	5562 (0.7)	4625 (0.6)	937 (0.8)	3365 (0.8)	2772 (0.8)	593 (1.0)
Non-Hispanic or non-Latino	568 905 (67.6)	504 778 (70.0)	64 127 (53.1)	295 299 (73.5)	261 477 (76.4)	33 822 (57.2)
Unknown	267 407 (31.8)	211 702 (29.4)	55 705 (46.1)	103 033 (25.6)	78 292 (22.9)	24 741 (41.8)
Age group, y^c^						
<35	110 563 (13.1)	90 957 (12.6)	19 606 (16.2)	69 415 (17.3)	57 113 (16.7)	12 302 (20.8)
35-54	186 821 (22.2)	157 675 (21.9)	29 146 (24.1)	101 965 (25.4)	86 337 (25.2)	15 628 (26.4)
55-74	360 293 (42.8)	315 089 (43.7)	45 204 (37.4)	163 526 (40.7)	142 692 (41.7)	20 834 (35.2)
≥75	184 197 (21.9)	157 384 (21.8)	26 813 (22.2)	66 791 (16.6)	56 399 (16.5)	10 392 (17.6)
Sex						
Men	374 975 (44.5)	324 026 (44.9)	50 949 (42.2)	180 545 (44.9)	155 752 (45.5)	24 793 (41.9)
Women	466 899 (55.5)	397 079 (55.1)	69 820 (57.8)	221 152 (55.1)	186 589 (54.5)	34 363 (58.1)
Highmark Health Member^c^	444 318 (52.8)	377 328 (52.3)	66 990 (55.5)	266 554 (66.4)	222 718 (65.1)	43 836 (74.1)
Overall SVI quintile^c^						
1 (Low)	258 706 (30.7)	229 814 (31.9)	28 892 (23.9)	125 503 (31.2)	111 228 (32.5)	14 275 (24.1)
2	262 955 (31.2)	228 630 (31.7)	34 325 (28.4)	126 094 (31.4)	109 089 (31.9)	17 005 (28.7)
3	166 791 (19.8)	139 836 (19.4)	26 955 (22.3)	81 906 (20.4)	68 269 (19.9)	13 637 (23.1)
4	100 068 (11.9)	81 568 (11.3)	18 500 (15.3)	46 335 (11.5)	37 397 (10.9)	8938 (15.1)
5 (High)	53 354 (6.4)	41 257 (5.7)	12 097 (10.0)	21 859 (5.4)	16 558 (4.8)	5301 (9.0)
Needs by domain^d^						
Financial resource	44 187 (6.6)	NA	44 187 (39.7)	25 233 (7.5)	NA	25 233 (45.2)
Housing instability	29 579 (4.0)	NA	29 579 (25.8)	14 736 (4.2)	NA	14 736 (26.4)
Food insecurity	22 464 (3.0)	NA	22 464 (19.7)	11 350 (3.2)	NA	11 350 (20.5)
Health literacy	29 622 (4.3)	NA	29 622 (26.6)	10 917 (3.3)	NA	10 917 (19.9)
Social connections	22 803 (3.5)	NA	22 803 (21.8)	12 475 (3.9)	NA	12 475 (24.3)
Transportation	10 988 (1.6)	NA	10 988 (10.0)	4375 (1.2)	NA	4375 (7.9)
Safety	11 627 (1.8)	NA	11 627 (11.0)	5803 (1.8)	NA	5803 (11.1)

Abbreviations: NA, not applicable; SVI, Social Vulnerability Index.

^a^ One assessment instance is randomly sampled for each unique respondent.

^b^ No further information available.

^c^ Indicates at time of social determinants of health assessment.

^d^ Rates of needs for each domain were based on assessments with completed responses for those domains only, so the denominator varies by domain.

**Figure 1.**
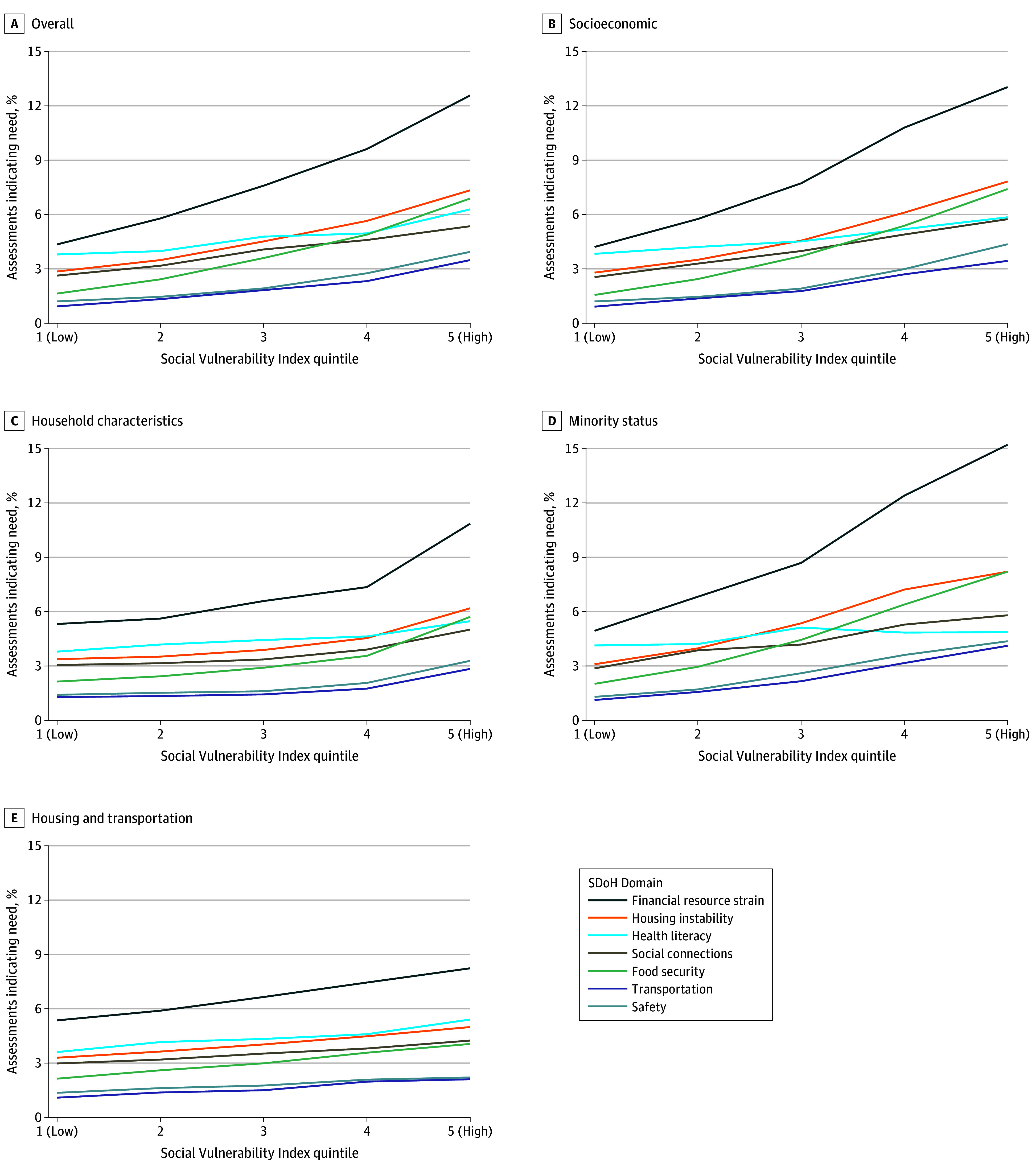
Rates of Positive Screens for Social Determinants of Health (SDoH) Needs by Domain and Social Vulnerability Index

Across all domains, increasing social vulnerability was associated with higher positivity rates in the SDoH assessment; in total, 11.2% of those residing in the lowest-risk quintile for overall SVI reported a need compared with 22.7% among those residing in the highest-risk quintile (Figure 1).^12,13,14,15,16,17,18^ However, the strength of the associations varied by domain. Financial resource strain, food insecurity, transportation access, and safety had the strongest association with SVI. Health literacy and social connections had the weakest associations with SVI. While absolute percentage point differences in positivity between low and high SVI were especially large for more prevalent needs like financial resource strain, smaller percentage point differences for less prevalent needs like transportation and safety translated to large relative increases in positivity. Associations also varied considerably across the 4 individual SVI themes. Overall, socioeconomic and racial and ethnic minority status themes were more strongly associated with positive assessments for all SDoH needs than household characteristics or housing type and transportation themes. Results from logistic regression models (Table 3) largely mirrored bivariate findings. Even after adjusting for covariates, odds of a positive assessment were significantly higher with increasing SVI for all combinations of outcome and SVI theme. There were clear differences in the strength of the various SVI scores. The socioeconomic theme had the strongest association with 6 SDoH domains (range of adjusted ORs [AORs], 1.70 [95% CI, 1.58-1.84] to 4.03 [95% CI, 3.75-4.34]) and had a stronger association with every domain than did the composite SVI. The racial and ethnic minority status theme was most strongly associated with housing instability (AOR, 2.37 [95% CI, 2.20-2.56]) and more strongly assocated with all 7 outcomes than with household characteristics and housing type and transportation themes.

**Table 3.** Results of Logistic Regression Models Estimating Risk for Positive Screening for Social Determinants of Health Needs by Domain as a Function of Social Vulnerability Index Quintile (1-5)

SVI Type	Mean expected increase in odds of a positive screen after 1-U increase in SVI quintile		Quintile 5 vs 1, AOR (95% CI)^a^^,^^b^
	OR (95% CI)	AOR (95% CI)^a^	
**Financial resource strain**			
Overall	1.35 (1.34-1.37)	1.25 (1.24-1.27)	2.64 (2.51-2.78)
Socioeconomic	1.41 (1.39-1.42)	1.27 (1.26-1.29)	2.69 (2.55-2.84)
Household characteristics	1.21 (1.20-1.22)	1.15 (1.13-1.16)	1.81 (1.73-1.89)
Racial and ethnic minority status	1.40 (1.38-1.41)	1.24 (1.23-1.26)	2.54 (2.38-2.70)
Housing type and transportation	1.11 (1.1-1.12)	1.11 (1.09-1.12)	1.52 (1.45-1.60)
**Housing instability**			
Overall	1.28 (1.27-1.3)	1.20 (1.19-1.22)	2.23 (2.09-2.38)
Socioeconomic	1.33 (1.31-1.34)	1.22 (1.20-1.24)	2.24 (2.10-2.40)
Household characteristics	1.17 (1.16-1.19)	1.12 (1.11-1.14)	1.63 (1.54-1.73)
Racial and ethnic minority status	1.34 (1.32-1.35)	1.22 (1.20-1.24)	2.37 (2.20-2.56)
Housing type and transportation	1.09 (1.07-1.10)	1.08 (1.07-1.10)	1.35 (1.27-1.43)
**Food insecurity**			
Overall	1.47 (1.45-1.49)	1.37 (1.35-1.39)	3.73 (3.48-4.00)
Socioeconomic	1.53 (1.51-1.55)	1.39 (1.37-1.41)	4.03 (3.75-4.34)
Household characteristics	1.29 (1.27-1.31)	1.22 (1.21-1.24)	2.36 (2.21-2.51)
Racial and ethnic minority status	1.49 (1.47-1.51)	1.33 (1.31-1.35)	3.39 (3.14-3.67)
Housing type and transportation	1.15 (1.13-1.17)	1.14 (1.12-1.16)	1.74 (1.62-1.86)
**Social connections**			
Overall	1.24 (1.22-1.26)	1.13 (1.12-1.15)	1.69 (1.57-1.82)
Socioeconomic	1.27 (1.25-1.29)	1.13 (1.12-1.15)	1.70 (1.58-1.84)
Household characteristics	1.14 (1.13-1.16)	1.07 (1.06-1.09)	1.34 (1.26-1.43)
Racial and ethnic minority status	1.26 (1.24-1.28)	1.11 (1.09-1.13)	1.55 (1.41-1.70)
Housing type and transportation	1.09 (1.07-1.10)	1.08 (1.06-1.09)	1.37 (1.28-1.46)
**Health literacy**			
Overall	1.14 (1.12-1.16)	1.11 (1.09-1.13)	1.67 (1.54-1.82)
Socioeconomic	1.15 (1.13-1.17)	1.12 (1.10-1.14)	1.74 (1.59-1.90)
Household characteristics	1.09 (1.08-1.11)	1.06 (1.04-1.07)	1.33 (1.24-1.43)
Racial and ethnic minority status	1.03 (1.02-1.05)	1.07 (1.05-1.09)	1.50 (1.33-1.69)
Housing type and transportation	1.08 (1.07-1.10)	1.06 (1.04-1.07)	1.25 (1.17-1.35)
**Transportation**			
Overall	1.36 (1.32-1.39)	1.29 (1.26-1.32)	2.97 (2.65-3.32)
Socioeconomic	1.40 (1.37-1.43)	1.31 (1.28-1.34)	3.05 (2.71-3.42)
Household characteristics	1.18 (1.15-1.21)	1.14 (1.11-1.16)	1.79 (1.62-1.97)
Racial and ethnic minority status	1.38 (1.35-1.41)	1.29 (1.26-1.32)	2.80 (2.47-3.19)
Housing type and transportation	1.15 (1.12-1.17)	1.14 (1.11-1.17)	1.68 (1.51-1.86)
**Safety**			
Overall	1.35 (1.33-1.38)	1.28 (1.25-1.31)	2.94 (2.68-3.23)
Socioeconomic	1.39 (1.36-1.42)	1.29 (1.27-1.32)	3.01 (2.73-3.32)
Household characteristics	1.22 (1.20-1.25)	1.18 (1.15-1.20)	2.08 (1.90-2.26)
Racial and ethnic minority status	1.39 (1.36-1.42)	1.29 (1.26-1.32)	2.85 (2.55-3.19)
Housing type and transportation	1.12 (1.09-1.14)	1.11 (1.09-1.13)	1.50 (1.37-1.65)

Abbreviations: AOR, adjusted odds ratio (OR); SVI, Social Vulnerability Index.

^a^ Adjusted for age, sex, and assessment source. Full model results are available in eTables 2 to 7 in Supplement 1.

^b^ Quintile 5 is the highest and quintile 1 the lowest quintile.

Even among the consistently stronger SVI estimates, associations varied greatly by SDoH domain. By a large margin, food insecurity was most closely associated with SVI. On average, risk for food insecurity increased by 39% with each quintile increase in socioeconomic SVI, and odds were 300% higher among those in the highest vs the lowest risk quintile (AOR, 4.03 [95% CI, 3.75-4.34]; AOR range, 1.74 [95% CI, 1.62-1.86] to 3.73 [95% CI, 3.48-4.00] for the other SVI measures). Financial resource strain, housing instability, transportation, and safety all had positive associations with overall, socioeconomic, and racial and ethnic minority status SVI, with AORs ranging from 2.23 (95% CI, 2.09-2.38) to 3.05 (95% CI, 2.71-3.42).

Results of generalized additive models suggest that some degree of nonlinearity in the association between SVI and positive SDoH screening was typical and easily detected in our very large sample. eFigures 1 to 2 and eTables 2 to 7 in Supplement 1 provide full model information, including parametric coefficients and smoothed terms, partial effects smooth plots, and ORs calculated across the SVI distribution. These models highlight notable themes regarding the specific shape of the positive relationship between SVI and positive responses. For overall and socioeconomic SVI, the largest increases in risk were seen at the low and high end of the SVI range, particularly from the 80th to 100th risk percentile. The racial and ethnic minority status theme was generally associated with larger increases in risk over each increasing quintile. The household characteristics theme had the most nonlinearity of all SVI measures, in part due to a consistently steep increase in risk over the top 20% of the SVI distribution.

**Figure 2.**
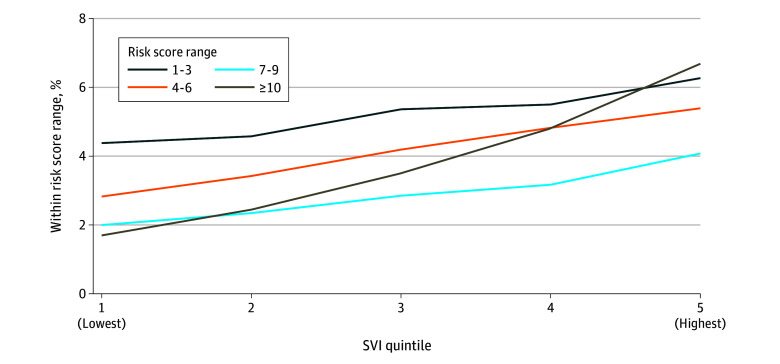
Association of Cumulative Social Determinants of Health and Social Vulnerability Index (SVI) Risks Composite scores of 10 or greater indicate greater aggregate severity and complexity of social determinants of health needs.

The severity and complexity of assessed needs was also positively associated with overall SVI (Figure 2). A total of 4382 assessments from individuals living in the lowest quintile SVI areas (1.7%) had a composite score at or above 10, while 3567 assessments from individuals living in the highest quintile SVI areas (6.7%) met or exceeded a composite risk score of 10.

## Discussion

We found that social needs identified through the broad deployment of an SDoH assessment tool correlated with a geographically based community vulnerability index.^4^ This was true for domain-specific SDoH needs and for composite scores representing overall severity and complexity of SDoH needs. As hypothesized, the strength of the associations varied across the 4 SVI themes. Individual SDoH needs were not necessarily most closely associated with the corresponding SVI theme.

There are important practical implications for these findings. Appropriate provision of limited resources for addressing social needs is often complicated by the lack of individual-level data. As a result, area-level measures like SVI are commonly used to guide targeting, despite little evidence linking these measures to self-reported needs. While these findings confirmed a general positive association between SDoH and SVI, the socioeconomic and racial and ethnic minority status SVI themes were more closely associated with the full range of SDoH needs than other themes. These themes likely reflect broader societal and historical factors, including longstanding laws and policy decisions such as redlining and unfair lending practices that contribute to ongoing systemic and structural racism. Notably, among study participants with race and ethnicity data available, only approximately 7.5% identified as Black or Hispanic. Thus, racial and ethnic minority status SVI may reflect not only the direct consequences of structural racism on members of marginalized racial and ethnic minority groups, but also effects of the resulting inequitable distribution of resources on communities more broadly. Self-reported SDoH needs related to health literacy and social connections had a much weaker association with SVI measures than needs in other domains, suggesting that alternative approaches to risk stratification may be required for these domains. Finally, although the odds of positive screening for 1 or more SDoH needs were up to 3 times higher among those in the highest quintile of SVI risk compared with the lowest, more than 3 in 4 individuals in the highest risk group reported no SDoH need. Despite the COVID-19 pandemic, SDoH identification rates remained relatively constant over 40 months of follow-up, never deviating more than 1.2% in any year.

### Strengths and Limitations

This study has several strengths. First, we are unaware of any prior reports in which a cohort of this size was used to compare an SDoH assessment tool with the often-cited census-based SVI. Second, several features of the study strengthen generalizability: the cohort was diverse and included individuals with all insurance statuses and types, and individuals were assessed in a variety of settings and modalities. Third, self-reported SDoH needs were captured using a standard instrument consisting of validated questions.

This study also has several limitations. While self-reporting is the gold standard for capturing SDoH needs, the true extent of needs may be underreported due to distrust of the health care system, stigma, or fear of negative consequences, which may vary by SVI. Additionally, assessments capture a snapshot of needs. This may lead to underreporting of needs, as they are often episodic in nature. While in aggregate this population had a below average SVI risk, there were still several thousand individuals in every SVI quintile, allowing for high-powered comparisons across all levels. Because most respondents engaged with the assessment during a clinical encounter or through a payer program related to clinical factors, the assessed population tended to be biased toward those who use medical services. Notably, for identifying or intervening in issues closely related to limited access to medical services, measures like SVI that do not contain this bias may be more suitable. We did not assess comorbidities, so it is unknown whether differences in health status by SVI may contribute to the higher rate of SDoH needs among those in highly vulnerable neighborhoods. However, the association between SVI and SDoH needs in regression models was robust to age, sex, and assessment source, which likely captures some variability related to health status. The study was also limited by unavailable or incomplete data for race and ethnicity, language, income, and educational level.

## Conclusions

In this cross-sectional study of self-reported SDoH needs and their association with community-level social vulnerability measures, individuals who lived in high SVI areas were more likely to have 1 or more SDoH issues and were far more likely to have significant needs across multiple domains. These findings cross-validate both assessment tools and underscore the interrelated nature and relevance of both personal and community context in social needs.^19,20,21,22,23^ A more complete understanding of risk and protective factors at both levels, including their interactions, can inform targeted and cross-sector interventions to significantly affect health care outcomes. We believe that using person-level SDoH data in conjunction with area-level data will allow clinicians, health systems, scientists, and payers to advance clinical outcomes and health equity through more cost-effective outreach efforts.

Future studies should analyze differences by individual factors like sociodemographic characteristics and health status, as well as other community-level factors like rurality and treatment access, to help identify meaningful heterogeneity in patterns of SDoH needs. Such investigations may inform the development of more tailored interventions and improve targeted outreach and services. Additionally, future research leveraging longitudinal assessment data may elucidate need patterns over time and the factors that contribute to or hinder SDoH gap closure and maintenance. Finally, predictive models trained on existing assessment results and other associated data points may be leveraged to identify individuals at high risk for SDoH needs who have not yet been assessed.

## Notes

Supplement 1. **eFigure 1.** GAM Partial Effects Smooth Plots Illustrating Nonlinear Associations Between SVI and Assessment Responses (Faceted by Assessment Response Domain) **eFigure 2.** GAM Partial Effects Smooth Plots Illustrating Nonlinear Associations Between SVI and Assessment Responses (Faceted by SVI) **eTable 1**. Scoring Rubric for Composite Social Determinants of Health Risk **eTable 2.** GAM-Model Estimated Adjusted Odds Ratios for SDoH Needs Calculated Across the Range of Each SVI Quintile **eTable 3.** Results of GAM Models Estimating Risk for Positive Assessment by Domain as a Function of Overall SVI **eTable 4.** Results of GAM Models Estimating Risk for Positive Assessment by Domain as a Function of Socioeconomic SVI **eTable 5.** Results of GAM Models Estimating Risk for Positive Assessment by Domain as a Function of Household Characteristics SVI **eTable 6.** Results of GAM Models Estimating Risk for Positive Assessment by Domain as a Function of Minority Status SVI **eTable 7.** Results of GAM Models Estimating Risk for Positive Assessment by SDoH Domain as a Function of Housing and Transportation SVI

Supplement 2.
Data Sharing Statement
