# Supplementary material for: Self-Reported Social Determinants of Health and Area-Level Social Vulnerability
Source: JAMA Netw Open. 2024 May 20;7(5):e2412109. doi: 10.1001/jamanetworkopen.2024.12109 (PMC11107301; doi:10.1001/jamanetworkopen.2024.12109)
Supplement: Supplement 1. — eFigure 1. GAM Partial Effects Smooth Plots Illustrating Nonlinear Associations Between SVI and Assessment Responses (Faceted by Assessment Response Domain) eFigure 2. GAM Partial Effects Smooth Plots Illustrating Nonlinear Associations Between SVI and Assessment Responses (Faceted by SVI) eTable 1. Scoring Rubric for Composite Social Determinants of Health Risk eTable 2. GAM-Model Estimated Adjusted Odds Ratios for SDoH Needs Calculated Across the Range of Each SVI Quintile eTable 3. Results of GAM Models Estimating Risk for Positive Assessment by Domain as a Function of Overall SVI eTable 4. Results of GAM Models Estimating Risk for Positive Assessment by Domain as a Function of Socioeconomic SVI eTable 5. Results of GAM Models Estimating Risk for Positive Assessment by Domain as a Function of Household Characteristics SVI eTable 6. Results of GAM Models Estimating Risk for Positive Assessment by Domain as a Function of Minority Status SVI eTable 7. Results of GAM Models Estimating Risk for Positive Assessment by SDoH Domain as a Function of Housing and Transportation SVI [file jamanetwopen-e2412109-s001.pdf]

Supplementary Online Content

Brignone E, LeJeune K, Mihalko AE, Shannon AL, Sinoway LI. Self-reported social determinants of health and area-level social vulnerability. *JAMA Netw Open*. 2024;7(5):e2412109. doi:10.1001/jamanetworkopen.2024.12109

eFigure 1. GAM Partial Effects Smooth Plots Illustrating Nonlinear Associations Between SVI and Assessment Responses (Faceted by Assessment Response Domain)

eFigure 2. GAM Partial Effects Smooth Plots Illustrating Nonlinear Associations Between SVI and Assessment Responses (Faceted by SVI)

eTable 1. Scoring Rubric for Composite Social Determinants of Health Risk

eTable 2. GAM-Model Estimated Adjusted Odds Ratios for SDoH Needs Calculated Across the Range of Each SVI Quintile

eTable 3. Results of GAM Models Estimating Risk for Positive Assessment by Domain as a Function of Overall SVI

eTable 4. Results of GAM Models Estimating Risk for Positive Assessment by Domain as a Function of Socioeconomic SVI

eTable 5. Results of GAM Models Estimating Risk for Positive Assessment by Domain as a Function of Household Characteristics SVI

eTable 6. Results of GAM Models Estimating Risk for Positive Assessment by Domain as a Function of Minority Status SVI

eTable 7. Results of GAM Models Estimating Risk for Positive Assessment by SDoH Domain as a Function of Housing and Transportation SVI

This supplementary material has been provided by the authors to give readers additional information about their work.

© 2024 Brignone E et al. *JAMA Network Open*.

eFigure 1. GAM Partial Effects Smooth Plots Illustrating Nonlinear Associations Between SVI and Assessment Responses (Faceted by Assessment Response Domain)

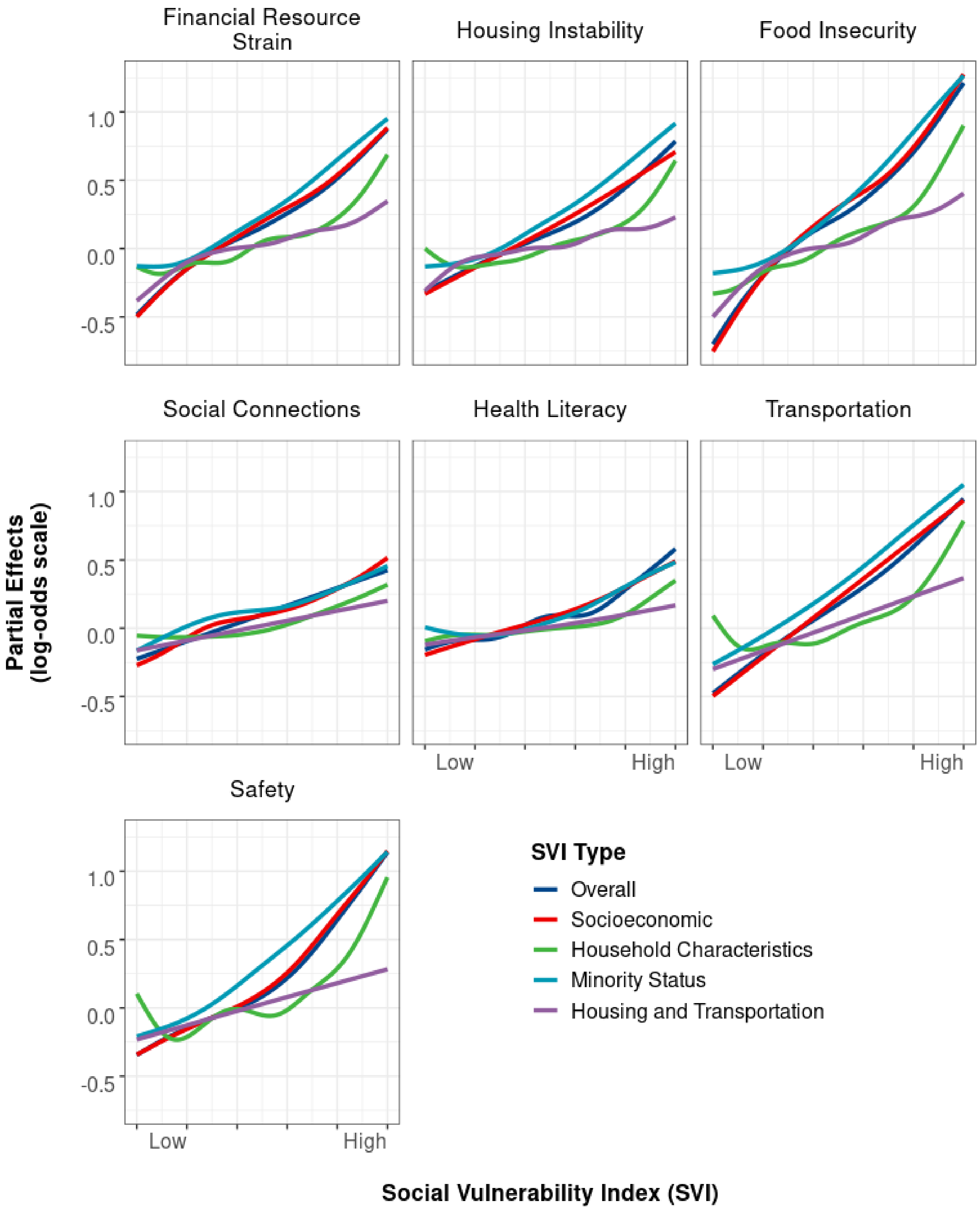

© 2024 Brignone E et al. *JAMA Network Open*.

eFigure 2. GAM Partial Effects Smooth Plots Illustrating Nonlinear Associations Between SVI and Assessment Responses (Faceted by SVI)

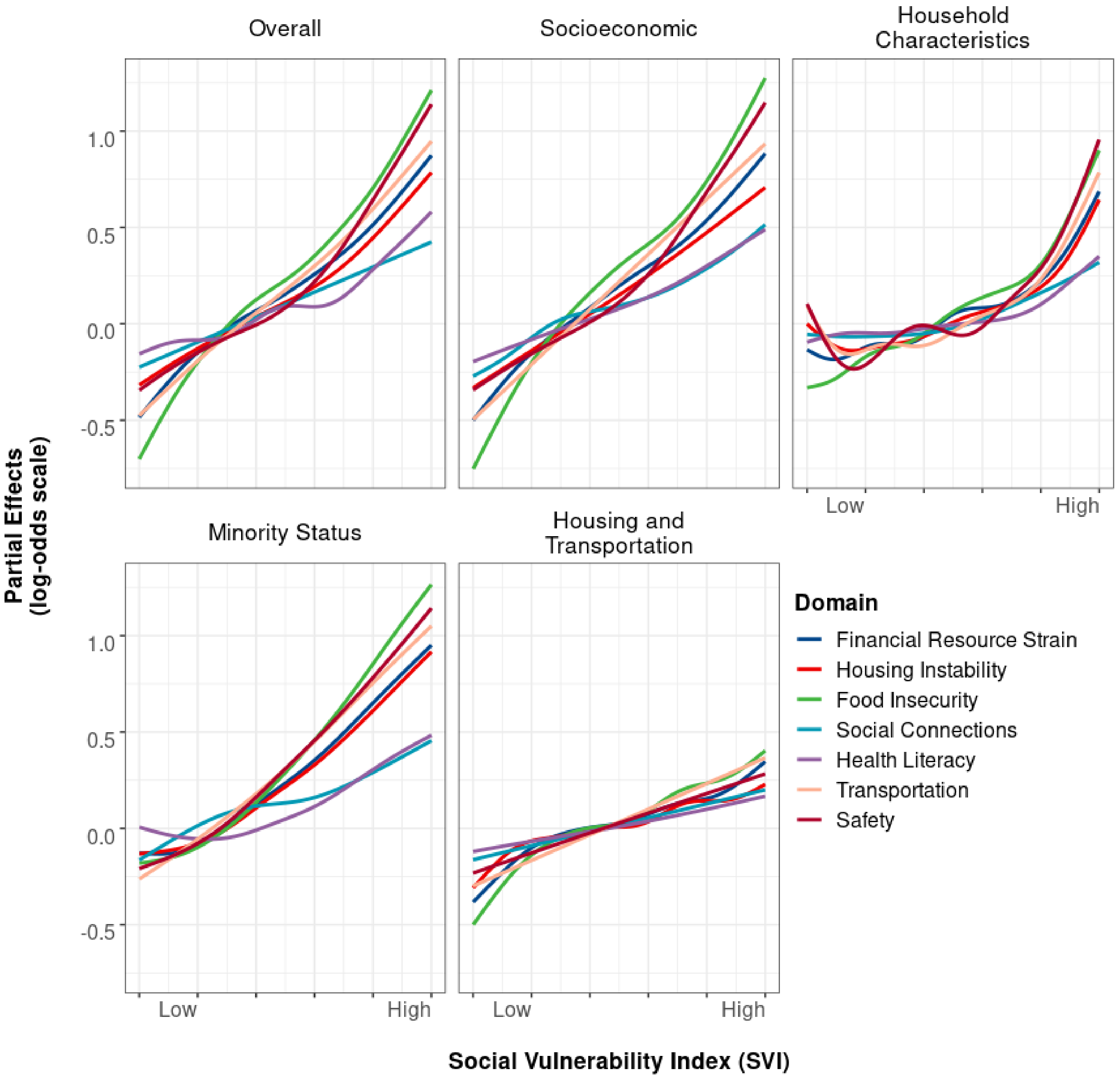

© 2024 Brignone E et al. *JAMA Network Open*.

eTable 1. Scoring Rubric for Composite Social Determinants of Health Risk[a]

Domain	Question	Answer Choice	Score
Social Connections	How often do you feel that you lack companionship?	Hardly ever	0
		Some of the time	0
		Often	1
	How often do you feel left out?	Hardly ever	0
		Some of the time	0
		Often	1
	How often do you feel isolated from others?	Hardly ever	0
		Some of the time	0
		Often	1
Financial Resource Strain	Sometimes people find that their income does not quite cover their living costs. In the last 12 months, has this happened to you?	Yes	6
		No	0
		Don't know	0
Health Literacy	How often do you need to have someone help you when you read instructions, pamphlets, or other written material from your doctor or pharmacist?	Never	0
		Rarely	0
		Sometimes	0
		Often	1
		Always	2
Food Insecurity	"Within the past 12 months, we worried whether our food would run out before we got the money to buy more."	Often true	5
		Sometimes true	3
		Never true	0
	"Within the past 12 months, the food we bought just didn't last and we didn't have money to get more."	Often true	7
		Sometimes true	5
		Never true	0
Transportation	Has a lack of transportation kept you from medical appointments, meetings, work, or from getting things needed for daily living? (check all that apply)	Yes, it has kept me from medical appointments or from getting my medications	7
		Yes, it has kept me from non-medical meetings, appointments, work, or from getting things that I need	4
		No	0
Safety	Do you feel safe in your neighborhood?	Yes	0
		No	2
	Are you afraid of anyone close to you?	Yes	7
		No	0
Housing Stability	In the past 2 months, have you been living in a stable housing that you own, rent, or stay in as part of a household?	Yes	0
		No	7
		Yes	5

© 2024 Brignone E et al. *JAMA Network Open*.

	In the next 2 months, are you worried you may not have stable	No	0
	In the past 12 months, has the electric, gas, oil, or water company threatened to shut off services in your home?	Yes	5
		No	0
		Already shut off	7

[a] Missing responses and those with a response of "I choose not to answer" were scored as 0.

© 2024 Brignone E et al. *JAMA Network Open*.

eTable 2. GAM-Model Estimated Adjusted Odds Ratios for SDoH Needs Calculated Across the Range of Each SVI Quintile

Domain	Q1	Q2	Q3	Q4	Q5
	Odds Ratio (95% CI)				
**Overall SVI**					
Financial Resource Strain	1.4 (1.27-1.55)	1.24 (1.18-1.31)	1.21 (1.14-1.28)	1.29 (1.21-1.38)	1.43 (1.24-1.65)
Housing Instability	1.21 (1.09-1.34)	1.18 (1.11-1.25)	1.17 (1.1-1.24)	1.29 (1.2-1.38)	1.4 (1.21-1.62)
Food Insecurity	1.65 (1.41-1.94)	1.38 (1.27-1.5)	1.26 (1.16-1.36)	1.42 (1.3-1.56)	1.66 (1.37-2.02)
Social Connections	1.14 (1.11-1.17)	1.14 (1.11-1.16)	1.14 (1.11-1.16)	1.14 (1.11-1.16)	1.14 (1.11-1.17)
Health Literacy	1.07 (0.92-1.25)	1.11 (1.02-1.21)	1.07 (0.97-1.18)	1.21 (1.08-1.36)	1.35 (1.02-1.77)
Transportation	1.33 (1.15-1.54)	1.28 (1.19-1.39)	1.28 (1.18-1.38)	1.35 (1.23-1.47)	1.42 (1.2-1.68)
Safety	1.22 (1.02-1.45)	1.16 (1.05-1.27)	1.25 (1.13-1.38)	1.53 (1.37-1.7)	1.64 (1.31-2.05)
**Socioeconomic SVI**					
Financial Resource Strain	1.42 (1.28-1.57)	1.26 (1.2-1.33)	1.24 (1.17-1.31)	1.27 (1.19-1.35)	1.41 (1.23-1.62)
Housing Instability	1.21 (1.14-1.29)	1.21 (1.17-1.25)	1.22 (1.18-1.27)	1.25 (1.19-1.3)	1.26 (1.17-1.36)
Food Insecurity	1.73 (1.46-2.06)	1.44 (1.32-1.56)	1.29 (1.18-1.4)	1.39 (1.26-1.52)	1.71 (1.4-2.08)
Social Connections	1.22 (1.07-1.39)	1.15 (1.07-1.23)	1.08 (1-1.16)	1.16 (1.07-1.27)	1.26 (1.04-1.52)
Health Literacy	1.12 (1.02-1.23)	1.11 (1.06-1.17)	1.12 (1.06-1.18)	1.17 (1.1-1.26)	1.21 (1.06-1.37)
Transportation	1.33 (1.28-1.38)	1.33 (1.28-1.38)	1.33 (1.28-1.38)	1.33 (1.28-1.38)	1.33 (1.28-1.38)
Safety	1.21 (1.01-1.43)	1.18 (1.07-1.29)	1.28 (1.16-1.41)	1.53 (1.38-1.7)	1.59 (1.28-1.97)
**Household Characteristics SVI**					
Financial Resource Strain	1.01 (0.88-1.17)	1.06 (0.98-1.14)	1.16 (1.07-1.25)	1.15 (1.06-1.25)	1.59 (1.37-1.84)
Housing Instability	0.88 (0.74-1.03)	1.07 (0.98-1.16)	1.14 (1.04-1.24)	1.14 (1.03-1.25)	1.57 (1.33-1.87)
Food Insecurity	1.17 (0.96-1.44)	1.13 (1.02-1.25)	1.21 (1.09-1.33)	1.19 (1.07-1.32)	1.81 (1.51-2.17)

© 2024 Brignone E et al. *JAMA Network Open*.

eTable 2. GAM-Model Estimated Adjusted Odds Ratios for SDoH Needs Calculated Across the Range of Each SVI Quintile

	Q1	Q2	Q3	Q4	Q5
Social Connections	0.99 (0.89-1.1)	1.02 (0.96-1.08)	1.08 (1.02-1.14)	1.14 (1.08-1.22)	1.17 (1.04-1.32)
Health Literacy	1.05 (0.9-1.23)	1.02 (0.95-1.1)	1.04 (0.96-1.12)	1.1 (1-1.19)	1.28 (1.08-1.52)
Transportation	0.8 (0.6-1.06)	1.02 (0.88-1.19)	1.17 (1-1.36)	1.21 (1.03-1.42)	1.74 (1.31-2.31)
Safety	0.73 (0.56-0.95)	1.23 (1.06-1.42)	0.99 (0.86-1.15)	1.35 (1.16-1.58)	1.95 (1.5-2.53)
**Minority Status SVI**					
Financial Resource Strain	1.04 (0.97-1.12)	1.24 (1.17-1.31)	1.26 (1.18-1.34)	1.35 (1.25-1.45)	1.35 (1.13-1.61)
Housing Instability	1.06 (0.97-1.15)	1.2 (1.13-1.28)	1.25 (1.16-1.34)	1.32 (1.22-1.44)	1.36 (1.13-1.63)
Food Insecurity	1.09 (0.98-1.2)	1.26 (1.17-1.36)	1.38 (1.27-1.5)	1.48 (1.36-1.63)	1.51 (1.24-1.84)
Social Connections	1.19 (1.09-1.31)	1.11 (1.04-1.18)	1.05 (0.97-1.13)	1.14 (1.04-1.25)	1.18 (0.97-1.44)
Health Literacy	0.94 (0.86-1.03)	1.05 (0.97-1.13)	1.13 (1.03-1.23)	1.21 (1.08-1.36)	1.19 (0.94-1.52)
Transportation	1.23 (1.11-1.36)	1.26 (1.18-1.35)	1.31 (1.22-1.41)	1.35 (1.23-1.49)	1.34 (1.15-1.58)
Safety	1.14 (1.01-1.28)	1.27 (1.17-1.38)	1.34 (1.23-1.47)	1.38 (1.24-1.54)	1.43 (1.16-1.78)
**Housing and Transportation SVI**					
Financial Resource Strain	1.32 (1.17-1.5)	1.11 (1.04-1.18)	1.07 (1-1.14)	1.09 (1.01-1.16)	1.21 (1.05-1.4)
Housing Instability	1.28 (1.09-1.49)	1.06 (0.98-1.15)	1.04 (0.96-1.13)	1.11 (1.02-1.22)	1.09 (0.9-1.31)
Food Insecurity	1.44 (1.2-1.72)	1.15 (1.05-1.26)	1.09 (1-1.19)	1.15 (1.05-1.27)	1.18 (0.98-1.44)
Social Connections	1.08 (1.05-1.1)	1.08 (1.05-1.1)	1.08 (1.05-1.1)	1.08 (1.05-1.1)	1.08 (1.05-1.1)
Health Literacy	1.05 (1-1.11)	1.05 (1.02-1.09)	1.06 (1.03-1.09)	1.06 (1.03-1.1)	1.07 (1.01-1.13)
Transportation	1.14 (1.1-1.18)	1.14 (1.1-1.18)	1.14 (1.1-1.18)	1.14 (1.1-1.18)	1.14 (1.1-1.18)
Safety	1.11 (1.07-1.15)	1.11 (1.07-1.15)	1.11 (1.07-1.14)	1.11 (1.07-1.14)	1.11 (1.07-1.15)

© 2024 Brignone E et al. *JAMA Network Open*.

eTable 3. Results of GAM Models Estimating Risk for Positive Assessment by Domain as a Function of Overall SVI

	**Dependent Variable: Financial Resource Strain**				
Component	Term	Estimate	SE	t-value	p-value
Parametric Coefficients	Intercept	-3.48	0.015	-238.143	<.001
	Female vs. Male	0.183	0.014	13.209	<.001
	Source: Telephonic Clinical Services (Payor)	1.379	0.015	89.589	<.001
	Source: Clinical Setting - Kiosk	1.276	0.031	41.578	<.001
	Source: Web-based	2.11	0.03	70.012	<.001
		edf	SE	F-value	p-value
Smooth Terms	s(SVI Overall Percentile)	4.568	5.611	1794.829	<.001
	s(Age)	7.772	8.457	2403.079	<.001
	**Dependent Variable: Housing Instability**				
		Estimate	SE	t-value	p-value
Parametric Coefficients	Intercept	-3.655	0.016	-227.67	<.001
	Female vs. Male	0.002	0.017	0.109	0.913
	Source: Telephonic Clinical Services (Payor)	1.079	0.018	58.382	<.001
	Source: Clinical Setting - Kiosk	0.755	0.042	17.797	<.001
	Source: Web-based	1.089	0.046	23.739	<.001
		edf	SE	F-value	p-value
Smooth Terms	s(SVI Overall Percentile)	3.699	4.592	795.913	<.001
	s(Age)	7.945	8.633	759.465	<.001
	**Dependent Variable: Food Insecurity**				
		Estimate	SE	t-value	p-value
Parametric Coefficients	Intercept	-4.406	0.021	-208.158	<.001
	Female vs. Male	0.258	0.02	12.766	<.001
	Source: Telephonic Clinical Services (Payor)	1.311	0.022	59.368	<.001
	Source: Clinical Setting - Kiosk	1.221	0.045	27.374	<.001
	Source: Web-based	2.214	0.038	57.567	<.001

© 2024 Brignone E et al. *JAMA Network Open*.

eTable 3. Results of GAM Models Estimating Risk for Positive Assessment by Domain as a Function of Overall SVI

		edf	SE	F-value	p-value
Smooth Terms	s(SVI Overall Percentile)	4.798	5.876	1724.517	<.001
	s(Age)	7.777	8.402	1205.076	<.001
		**Dependent Variable: Social Connections**			
		Estimate	SE	t-value	p-value
Parametric Coefficients	Intercept	-4.056	0.019	-209.681	<.001
	Female vs. Male	0.215	0.019	11.195	<.001
	Source: Telephonic Clinical Services (Payor)	1.435	0.021	68.545	<.001
	Source: Clinical Setting - Kiosk	0.888	0.046	19.385	<.001
	Source: Web-based	1.94	0.039	50.053	<.001
		edf	SE	F-value	p-value
Smooth Terms	s(SVI Overall Percentile)	1.006	1.012	287.581	<.001
	s(Age)	8.703	8.964	1092.553	<.001
		**Dependent Variable: Health Literacy**			
		Estimate	SE	t-value	p-value
Parametric Coefficients	Intercept	-4.043	0.02	-201.532	<.001
	Female vs. Male	-0.218	0.02	-10.719	<.001
	Source: Telephonic Clinical Services (Payor)	0.949	0.022	43.078	<.001
	Source: Clinical Setting - Kiosk	0.318	0.068	4.68	<.001
	Source: Web-based	0.087	0.087	0.999	0.318
		edf	SE	F-value	p-value
Smooth Terms	s(SVI Overall Percentile)	5.328	6.447	194.143	<.001
	s(Age)	8.794	8.986	12574.678	<.001
		**Dependent Variable: Transportation**			
		Estimate	SE	t-value	p-value
Parametric Coefficients	Intercept	-5.141	0.033	-158.176	<.001
	Female vs. Male	0.228	0.031	7.225	<.001

© 2024 Brignone E et al. *JAMA Network Open*.

eTable 3. Results of GAM Models Estimating Risk for Positive Assessment by Domain as a Function of Overall SVI

	Source: Telephonic Clinical Services (Payor)	1.002	0.034	29.32	<.001
	Source: Clinical Setting - Kiosk	1.219	0.069	17.675	<.001
	Source: Web-based	1.55	0.071	21.918	<.001
		edf	SE	F-value	p-value
Smooth Terms	s(SVI Overall Percentile)	2.597	3.253	474.393	<.001
	s(Age)	8.293	8.834	240.883	<.001
		**Dependent Variable: Safety**			
		Estimate	SE	t-value	p-value
Parametric Coefficients	Intercept	-4.551	0.026	-176.008	<.001
	Female vs. Male	0.214	0.027	7.793	<.001
	Source: Telephonic Clinical Services (Payor)	0.867	0.029	29.404	<.001
	Source: Clinical Setting - Kiosk	0.656	0.065	10.065	<.001
	Source: Web-based	1.281	0.06	21.478	<.001
		edf	SE	F-value	p-value
Smooth Terms	s(SVI Overall Percentile)	3.922	4.857	664.891	<.001
	s(Age)	7.809	8.549	84.07	<.001

Notes) Source reference level = Clinical Setting - Staff; Age variable is scaled

© 2024 Brignone E et al. *JAMA Network Open*.

eTable 4. Results of GAM Models Estimating Risk for Positive Assessment by Domain as a Function of Socioeconomic SVI

	**Dependent Variable: Financial Resource Strain**				
Component	Term	Estimate	SE	t-value	p-value
Parametric Coefficients	Intercept	-3.473	0.015	-237.7	<.001
	Female vs. Male	0.185	0.014	13.334	<.001
	Source: Telephonic Clinical Services (Payor)	1.349	0.016	86.969	<.001
	Source: Clinical Setting - Kiosk	1.273	0.031	41.452	<.001
	Source: Web-based	2.087	0.03	69.156	<.001
		edf	SE	F-value	p-value
Smooth Terms	s(SVI Socioeconomic Percentile)	4.489	5.518	1915.899	<.001
	s(Age)	7.735	8.432	2368.902	<.001
	**Dependent Variable: Housing Instability**				
		Estimate	SE	t-value	p-value
Parametric Coefficients	Intercept	-3.65	0.016	-227.42	<.001
	Female vs. Male	0.003	0.017	0.202	0.84
	Source: Telephonic Clinical Services (Payor)	1.052	0.019	56.443	<.001
	Source: Clinical Setting - Kiosk	0.752	0.042	17.748	<.001
	Source: Web-based	1.068	0.046	23.259	<.001
		edf	SE	F-value	p-value
Smooth Terms	s(SVI Socioeconomic Percentile)	2.018	2.532	838.066	<.001
	s(Age)	7.945	8.633	749.344	<.001
	**Dependent Variable: Food Insecurity**				
		Estimate	SE	t-value	p-value
Parametric Coefficients	Intercept	-4.404	0.021	-207.812	<.001
	Female vs. Male	0.26	0.02	12.857	<.001
	Source: Telephonic Clinical Services (Payor)	1.266	0.022	56.93	<.001
	Source: Clinical Setting - Kiosk	1.219	0.045	27.319	<.001
	Source: Web-based	2.18	0.039	56.569	<.001
		edf	SE	F-value	p-value
Smooth Terms	s(SVI Socioeconomic Percentile)	4.879	5.962	1872.255	<.001

© 2024 Brignone E et al. *JAMA Network Open*.

eTable 4. Results of GAM Models Estimating Risk for Positive Assessment by Domain as a Function of Socioeconomic SVI

	s(Age)	7.783	8.406	1190.269	<.001
**Dependent Variable: Social Connections**					
		Estimate	SE	t-value	p-value
Parametric Coefficients	Intercept	-4.053	0.019	-209.507	<.001
	Female vs. Male	0.216	0.019	11.248	<.001
	Source: Telephonic Clinical Services (Payor)	1.423	0.021	67.437	<.001
	Source: Clinical Setting - Kiosk	0.885	0.046	19.318	<.001
	Source: Web-based	1.931	0.039	49.761	<.001
		edf	SE	F-value	p-value
Smooth Terms	s(SVI Socioeconomic Percentile)	4.255	5.247	292.475	<.001
	s(Age)	8.704	8.964	1093.785	<.001
**Dependent Variable: Health Literacy**					
		Estimate	SE	t-value	p-value
Parametric Coefficients	Intercept	-4.04	0.02	-201.379	<.001
	Female vs. Male	-0.217	0.02	-10.68	<.001
	Source: Telephonic Clinical Services (Payor)	0.934	0.022	42.098	<.001
	Source: Clinical Setting - Kiosk	0.319	0.068	4.686	<.001
	Source: Web-based	0.076	0.087	0.88	0.379
		edf	SE	F-value	p-value
Smooth Terms	s(SVI Socioeconomic Percentile)	2.506	3.139	201.814	<.001
	s(Age)	8.795	8.986	12622.117	<.001
**Dependent Variable: Transportation**					
		Estimate	SE	t-value	p-value
Parametric Coefficients	Intercept	-5.135	0.032	-158.044	<.001
	Female vs. Male	0.23	0.031	7.298	<.001
	Source: Telephonic Clinical Services (Payor)	0.963	0.034	27.987	<.001
	Source: Clinical Setting - Kiosk	1.217	0.069	17.645	<.001
	Source: Web-based	1.52	0.071	21.472	<.001
		edf	SE	F-value	p-value

© 2024 Brignone E et al. *JAMA Network Open*.

eTable 4. Results of GAM Models Estimating Risk for Positive Assessment by Domain as a Function of Socioeconomic SVI

Smooth Terms	s(SVI Socioeconomic Percentile)	1.003	1.007	526.268	<.001
	s(Age)	8.289	8.833	246.241	<.001
		**Dependent Variable: Safety**			
		Estimate	SE	t-value	p-value
Parametric Coefficients	Intercept	-4.544	0.026	-175.862	<.001
	Female vs. Male	0.215	0.027	7.847	<.001
	Source: Telephonic Clinical Services (Payor)	0.834	0.03	28.016	<.001
	Source: Clinical Setting - Kiosk	0.649	0.065	9.969	<.001
	Source: Web-based	1.251	0.06	20.947	<.001
		edf	SE	F-value	p-value
Smooth Terms	s(SVI Socioeconomic Percentile)	3.741	4.64	691.545	<.001
	s(Age)	7.815	8.553	81.041	<.001

Notes) Source reference level = Clinical Setting - Staff; Age variable is scaled

© 2024 Brignone E et al. *JAMA Network Open*.

eTable 5. Results of GAM Models Estimating Risk for Positive Assessment by Domain as a Function of Household Characteristics SVI

	**Dependent Variable: Financial Resource Strain**				
Component	Term	Estimate	SE	t-value	p-value
Parametric Coefficients	Intercept	-3.483	0.015	-238.749	<.001
	Female vs. Male	0.185	0.014	13.358	<.001
	Source: Telephonic Clinical Services (Payor)	1.439	0.015	94.159	<.001
	Source: Clinical Setting - Kiosk	1.258	0.031	41.042	<.001
	Source: Web-based	2.132	0.03	71.025	<.001
		edf	SE	F-value	p-value
Smooth Terms	s(SVI Household Characteristics Percentile)	6.97	8.05	858.246	<.001
	s(Age)	7.833	8.498	2446.197	<.001
	**Dependent Variable: Housing Instability**				
		Estimate	SE	t-value	p-value
Parametric Coefficients	Intercept	-3.658	0.016	-227.99	<.001
	Female vs. Male	0.004	0.017	0.255	0.799
	Source: Telephonic Clinical Services (Payor)	1.128	0.018	61.515	<.001
	Source: Clinical Setting - Kiosk	0.737	0.042	17.39	<.001
	Source: Web-based	1.111	0.046	24.251	<.001
		edf	SE	F-value	p-value
Smooth Terms	s(SVI Household Characteristics Percentile)	6.064	7.225	426.018	<.001
	s(Age)	7.901	8.607	768.752	<.001
	**Dependent Variable: Food Insecurity**				
		Estimate	SE	t-value	p-value
Parametric Coefficients	Intercept	-4.392	0.021	-208.722	<.001
	Female vs. Male	0.261	0.02	12.926	<.001
	Source: Telephonic Clinical Services (Payor)	1.39	0.022	63.53	<.001
	Source: Clinical Setting - Kiosk	1.201	0.045	26.977	<.001
	Source: Web-based	2.245	0.038	58.673	<.001
		edf	SE	F-value	p-value

© 2024 Brignone E et al. *JAMA Network Open*.

eTable 5. Results of GAM Models Estimating Risk for Positive Assessment by Domain as a Function of Household Characteristics SVI

Smooth Terms	s(SVI Household Characteristics Percentile)	6.028	7.19	878.96	<.001
	s(Age)	7.722	8.365	1233.835	<.001
	**Dependent Variable: Social Connections**				
		Estimate	SE	t-value	p-value
Parametric Coefficients	Intercept	-4.059	0.019	-209.908	<.001
	Female vs. Male	0.217	0.019	11.303	<.001
	Source: Telephonic Clinical Services (Payor)	1.47	0.021	70.681	<.001
	Source: Clinical Setting - Kiosk	0.875	0.046	19.094	<.001
	Source: Web-based	1.955	0.039	50.489	<.001
		edf	SE	F-value	p-value
Smooth Terms	s(SVI Household Characteristics Percentile)	3.192	3.969	128.645	<.001
	s(Age)	8.699	8.963	1095.545	<.001
	**Dependent Variable: Health Literacy**				
		Estimate	SE	t-value	p-value
Parametric Coefficients	Intercept	-4.045	0.02	-201.891	<.001
	Female vs. Male	-0.215	0.02	-10.585	<.001
	Source: Telephonic Clinical Services (Payor)	0.974	0.022	44.507	<.001
	Source: Clinical Setting - Kiosk	0.307	0.068	4.52	<.001
	Source: Web-based	0.099	0.087	1.136	0.256
		edf	SE	F-value	p-value
Smooth Terms	s(SVI Household Characteristics Percentile)	4.18	5.15	77.895	<.001
	s(Age)	8.792	8.986	12565.796	<.001
	**Dependent Variable: Transportation**				
		Estimate	SE	t-value	p-value
Parametric Coefficients	Intercept	-5.137	0.032	-158.577	<.001
	Female vs. Male	0.231	0.031	7.337	<.001
	Source: Telephonic Clinical Services (Payor)	1.075	0.034	31.692	<.001
	Source: Clinical Setting - Kiosk	1.179	0.069	17.083	<.001

© 2024 Brignone E et al. *JAMA Network Open*.

eTable 5. Results of GAM Models Estimating Risk for Positive Assessment by Domain as a Function of Household Characteristics SVI

	Source: Web-based	1.582	0.071	22.404	<.001
		edf	SE	F-value	p-value
Smooth Terms	s(SVI Household Characteristics Percentile)	5.8	6.96	224.426	<.001
	s(Age)	8.305	8.84	240.859	<.001
	**Dependent Variable: Safety**				
		Estimate	SE	t-value	p-value
Parametric Coefficients	Intercept	-4.555	0.026	-176.234	<.001
	Female vs. Male	0.218	0.027	7.93	<.001
	Source: Telephonic Clinical Services (Payor)	0.928	0.029	31.779	<.001
	Source: Clinical Setting - Kiosk	0.64	0.065	9.818	<.001
	Source: Web-based	1.311	0.06	22.02	<.001
		edf	SE	F-value	p-value
Smooth Terms	s(SVI Household Characteristics Percentile)	6.628	7.76	435.551	<.001
	s(Age)	7.779	8.53	86.562	<.001

Notes) Source reference level = Clinical Setting - Staff; Age variable is scaled

© 2024 Brignone E et al. *JAMA Network Open*.

eTable 6. Results of GAM Models Estimating Risk for Positive Assessment by Domain as a Function of Minority Status SVI

	**Dependent Variable: Financial Resource Strain**				
Component	Term	Estimate	SE	t-value	p-value
Parametric Coefficients	Intercept	-3.464	0.015	-237.774	<.001
	Female vs. Male	0.179	0.014	12.894	<.001
	Source: Telephonic Clinical Services (Payor)	1.389	0.015	90.04	<.001
	Source: Clinical Setting - Kiosk	1.207	0.031	39.415	<.001
	Source: Web-based	2.099	0.03	69.749	<.001
		edf	SE	F-value	p-value
Smooth Terms	s(SVI Minority Status Percentile)	4.619	5.646	1467.502	<.001
	s(Age)	7.865	8.519	2141.914	<.001
	**Dependent Variable: Housing Instability**				
		Estimate	SE	t-value	p-value
Parametric Coefficients	Intercept	-3.646	0.016	-227.446	<.001
	Female vs. Male	-0.003	0.017	-0.194	0.846
	Source: Telephonic Clinical Services (Payor)	1.078	0.019	58.23	<.001
	Source: Clinical Setting - Kiosk	0.702	0.042	16.576	<.001
	Source: Web-based	1.08	0.046	23.524	<.001
		edf	SE	F-value	p-value
Smooth Terms	s(SVI Minority Status Percentile)	3.848	4.756	825.833	<.001
	s(Age)	8.007	8.67	664.677	<.001
	**Dependent Variable: Food Insecurity**				
		Estimate	SE	t-value	p-value
Parametric Coefficients	Intercept	-4.372	0.021	-208.307	<.001
	Female vs. Male	0.254	0.02	12.563	<.001
	Source: Telephonic Clinical Services (Payor)	1.329	0.022	60.001	<.001
	Source: Clinical Setting - Kiosk	1.128	0.044	25.355	<.001
	Source: Web-based	2.204	0.038	57.447	<.001
		edf	SE	F-value	p-value

© 2024 Brignone E et al. *JAMA Network Open*.

eTable 6. Results of GAM Models Estimating Risk for Positive Assessment by Domain as a Function of Minority Status SVI

Smooth Terms	s(SVI Minority Status Percentile)	3.977	4.909	1375.104	<.001
	s(Age)	7.857	8.455	1063.596	<.001
**Dependent Variable: Social Connections**					
		Estimate	SE	t-value	p-value
Parametric Coefficients	Intercept	-4.052	0.019	-209.464	<.001
	Female vs. Male	0.214	0.019	11.153	<.001
	Source: Telephonic Clinical Services (Payor)	1.447	0.021	68.998	<.001
	Source: Clinical Setting - Kiosk	0.842	0.046	18.396	<.001
	Source: Web-based	1.936	0.039	49.928	<.001
		edf	SE	F-value	p-value
Smooth Terms	s(SVI Minority Status Percentile)	3.583	4.441	196.013	<.001
	s(Age)	8.71	8.965	1061.858	<.001
**Dependent Variable: Health Literacy**					
		Estimate	SE	t-value	p-value
Parametric Coefficients	Intercept	-4.045	0.02	-201.617	<.001
	Female vs. Male	-0.216	0.02	-10.634	<.001
	Source: Telephonic Clinical Services (Payor)	0.972	0.022	44.351	<.001
	Source: Clinical Setting - Kiosk	0.293	0.068	4.314	<.001
	Source: Web-based	0.096	0.087	1.099	0.272
		edf	SE	F-value	p-value
Smooth Terms	s(SVI Minority Status Percentile)	3.488	4.321	81.246	<.001
	s(Age)	8.794	8.986	12569.843	<.001
**Dependent Variable: Transportation**					
		Estimate	SE	t-value	p-value
Parametric Coefficients	Intercept	-5.127	0.032	-158.041	<.001
	Female vs. Male	0.222	0.031	7.055	<.001
	Source: Telephonic Clinical Services (Payor)	1.006	0.034	29.358	<.001
	Source: Clinical Setting - Kiosk	1.13	0.069	16.403	<.001

© 2024 Brignone E et al. *JAMA Network Open*.

eTable 6. Results of GAM Models Estimating Risk for Positive Assessment by Domain as a Function of Minority Status SVI

	Source: Web-based	1.533	0.071	21.665	<.001
		edf	SE	F-value	p-value
Smooth Terms	s(SVI Minority Status Percentile)	2.176	2.722	453.952	<.001
	s(Age)	8.332	8.851	267.294	<.001
	**Dependent Variable: Safety**				
		Estimate	SE	t-value	p-value
Parametric Coefficients	Intercept	-4.537	0.026	-175.817	<.001
	Female vs. Male	0.208	0.027	7.578	<.001
	Source: Telephonic Clinical Services (Payor)	0.858	0.03	28.962	<.001
	Source: Clinical Setting - Kiosk	0.582	0.065	8.94	<.001
	Source: Web-based	1.264	0.06	21.194	<.001
		edf	SE	F-value	p-value
Smooth Terms	s(SVI Minority Status Percentile)	3.104	3.863	607.689	<.001
	s(Age)	7.925	8.622	78.92	<.001

Notes) Source reference level = Clinical Setting - Staff; Age variable is scaled

© 2024 Brignone E et al. *JAMA Network Open*.

eTable 7. Results of GAM Models Estimating Risk for Positive Assessment by SDoH Domain as a Function of Housing and Transportation SVI

	**Dependent Variable: Financial Resource Strain**				
Component	Term	Estimate	SE	t-value	p-value
Parametric Coefficients	Intercept	-3.49	0.015	-239.14	<.001
	Female vs. Male	0.188	0.014	13.637	<.001
	Source: Telephonic Clinical Services (Payor)	1.474	0.015	97.105	<.001
	Source: Clinical Setting - Kiosk	1.23	0.031	40.208	<.001
	Source: Web-based	2.149	0.03	71.661	<.001
		edf	SE	F-value	p-value
Smooth Terms	s(SVI Housing and Transportation Percentile)	5.465	6.61	444.297	<.001
	s(Age)	7.788	8.467	2495.474	<.001
	**Dependent Variable: Housing Instability**				
		Estimate	SE	t-value	p-value
Parametric Coefficients	Intercept	-3.661	0.016	-228.207	<.001
	Female vs. Male	0.008	0.017	0.446	0.656
	Source: Telephonic Clinical Services (Payor)	1.157	0.018	63.595	<.001
	Source: Clinical Setting - Kiosk	0.716	0.042	16.913	<.001
	Source: Web-based	1.126	0.046	24.589	<.001
		edf	SE	F-value	p-value
Smooth Terms	s(SVI Housing and Transportation Percentile)	5.696	6.855	161.837	<.001
	s(Age)	7.915	8.615	794.357	<.001
	**Dependent Variable: Food Insecurity**				
		Estimate	SE	t-value	p-value
Parametric Coefficients	Intercept	-4.393	0.021	-208.841	<.001
	Female vs. Male	0.267	0.02	13.246	<.001
	Source: Telephonic Clinical Services (Payor)	1.444	0.022	66.484	<.001
	Source: Clinical Setting - Kiosk	1.149	0.044	25.874	<.001
	Source: Web-based	2.267	0.038	59.376	<.001
		edf	SE	F-value	p-value
Smooth Terms	s(SVI Housing and Transportation Percentile)	5.283	6.415	368.901	<.001

© 2024 Brignone E et al. *JAMA Network Open*.

eTable 7. Results of GAM Models Estimating Risk for Positive Assessment by SDoH Domain as a Function of Housing and Transportation SVI

	s(Age)	7.743	8.378	1257.886	<.001
	**Dependent Variable: Social Connections**				
		Estimate	SE	t-value	p-value
Parametric Coefficients	Intercept	-4.064	0.019	-210.075	<.001
	Female vs. Male	0.219	0.019	11.385	<.001
	Source: Telephonic Clinical Services (Payor)	1.486	0.021	71.972	<.001
	Source: Clinical Setting - Kiosk	0.862	0.046	18.852	<.001
	Source: Web-based	1.965	0.039	50.75	<.001
		edf	SE	F-value	p-value
Smooth Terms	s(SVI Housing and Transportation Percentile)	1.003	1.007	104.405	<.001
	s(Age)	8.702	8.963	1096.847	<.001
	**Dependent Variable: Health Literacy**				
		Estimate	SE	t-value	p-value
Parametric Coefficients	Intercept	-4.046	0.02	-202.113	<.001
	Female vs. Male	-0.215	0.02	-10.545	<.001
	Source: Telephonic Clinical Services (Payor)	0.984	0.022	45.197	<.001
	Source: Clinical Setting - Kiosk	0.297	0.068	4.374	<.001
	Source: Web-based	0.103	0.087	1.191	0.234
		edf	SE	F-value	p-value
Smooth Terms	s(SVI Housing and Transportation Percentile)	1.488	1.831	54.434	<.001
	s(Age)	8.793	8.986	12520.013	<.001
	**Dependent Variable: Transportation**				
		Estimate	SE	t-value	p-value
Parametric Coefficients	Intercept	-5.142	0.032	-158.689	<.001
	Female vs. Male	0.234	0.031	7.438	<.001
	Source: Telephonic Clinical Services (Payor)	1.101	0.034	32.7	<.001
	Source: Clinical Setting - Kiosk	1.157	0.069	16.82	<.001
	Source: Web-based	1.598	0.071	22.64	<.001
		edf	SE	F-value	p-value

© 2024 Brignone E et al. *JAMA Network Open*.

eTable 7. Results of GAM Models Estimating Risk for Positive Assessment by SDoH Domain as a Function of Housing and Transportation SVI

Smooth Terms	s(SVI Housing and Transportation Percentile)	1.006	1.013	130.29	<.001
	s(Age)	8.3	8.837	227.944	<.001
		**Dependent Variable: Safety**			
		Estimate	SE	t-value	p-value
Parametric Coefficients	Intercept	-4.551	0.026	-176.387	<.001
	Female vs. Male	0.221	0.027	8.075	<.001
	Source: Telephonic Clinical Services (Payor)	0.975	0.029	33.712	<.001
	Source: Clinical Setting - Kiosk	0.609	0.065	9.37	<.001
	Source: Web-based	1.334	0.059	22.445	<.001
		edf	SE	F-value	p-value
Smooth Terms	s(SVI Housing and Transportation Percentile)	1.074	1.145	101.375	<.001
	s(Age)	7.799	8.542	90.703	<.001

Notes) Source reference level = Clinical Setting - Staff; Age variable is scaled

© 2024 Brignone E et al. *JAMA Network Open*.
