# Supplementary material for: Self-Reported Social Determinants of Health and Area-Level Social Vulnerability
Source: JAMA Netw Open. 2024 May 20;7(5):e2412109. doi: 10.1001/jamanetworkopen.2024.12109 (PMC11107301; doi:10.1001/jamanetworkopen.2024.12109)
Supplement: Supplement 2. — Data Sharing Statement [file jamanetwopen-e2412109-s002.pdf]

Data Sharing Statement

Brignone. Self-Reported Social Determinants of Health and Area-Level Social Vulnerability. *JAMA Netw Open*. Published May 20, 2024. doi:10.1001/jamanetworkopen.2024.12109

Data
Data available: No

Additional Information
Explanation for why data not available: Due to the sensitivity of the data and legal and institutional requirements for the protection of health information, the analytic dataset will not be shared. Individuals requests for limited data may be considered on a case-by-case basis.
